# Correlation of *Klebsiella pneumoniae* Comparative Genetic Analyses with Virulence Profiles in a Murine Respiratory Disease Model

**DOI:** 10.1371/journal.pone.0107394

**Published:** 2014-09-09

**Authors:** Ramy A. Fodah, Jacob B. Scott, Hok-Hei Tam, Pearlly Yan, Tia L. Pfeffer, Ralf Bundschuh, Jonathan M. Warawa

**Affiliations:** 1 Department of Microbiology and Immunology, University of Louisville, Louisville, Kentucky, United States of America; 2 Center for Predictive Medicine, University of Louisville, Louisville, Kentucky, United States of America; 3 Dental School, University of Louisville, Louisville, Kentucky, United States of America; 4 Department of Chemical Engineering, Massachusetts Institute of Technology, Cambridge, Massachusetts, United States of America; 5 College of Dentistry, Ohio State University, Columbus, Ohio, United States of America; 6 The Comprehensive Cancer Center and The James Cancer Hospital and Solove Research Institute, Division of Hematology, Department of Internal Medicine, Ohio State University, Columbus, Ohio, United States of America; 7 Departments of Physics and Chemistry & Biochemistry and Center for RNA Biology, Ohio State University, Columbus, Ohio, United States of America; Quuen's University Belfast, United Kingdom

## Abstract

*Klebsiella pneumoniae* is a bacterial pathogen of worldwide importance and a significant contributor to multiple disease presentations associated with both nosocomial and community acquired disease. ATCC 43816 is a well-studied *K. pneumoniae* strain which is capable of causing an acute respiratory disease in surrogate animal models. In this study, we performed sequencing of the ATCC 43816 genome to support future efforts characterizing genetic elements required for disease. Furthermore, we performed comparative genetic analyses to the previously sequenced genomes from NTUH-K2044 and MGH 78578 to gain an understanding of the conservation of known virulence determinants amongst the three strains. We found that ATCC 43816 and NTUH-K2044 both possess the known virulence determinant for yersiniabactin, as well as a Type 4 secretion system (T4SS), CRISPR system, and an acetonin catabolism locus, all absent from MGH 78578. While both NTUH-K2044 and MGH 78578 are clinical isolates, little is known about the disease potential of these strains in cell culture and animal models. Thus, we also performed functional analyses in the murine macrophage cell lines RAW264.7 and J774A.1 and found that MGH 78578 (K52 serotype) was internalized at higher levels than ATCC 43816 (K2) and NTUH-K2044 (K1), consistent with previous characterization of the antiphagocytic properties of K1 and K2 serotype capsules. We also examined the three *K. pneumoniae* strains in a novel BALB/c respiratory disease model and found that ATCC 43816 and NTUH-K2044 are highly virulent (LD_50_<100 CFU) while MGH 78578 is relatively avirulent.

## Introduction

*Klebsiella pneumoniae* ssp. *pneumoniae* (*K. pneumoniae*) is responsible for emerging infectious disease and is a causative agent of both nosocomial and community acquired pneumonia (CAP) worldwide. The epidemiology of *K. pneumoniae* is complex, involving ecological persistence as well as carriage in both animal and human populations [1]. Carriage of *K. pneumoniae* is frequently associated with colonization of the upper respiratory tract or gastrointestinal (GI) tract, with the potential for GI tract amplification of antibiotic resistant strains of *K. pneumoniae* following antibiotic therapies [2]. *K. pneumoniae* opportunistically infects a variety of mucosal surfaces with the primary sites of infection including the urinary tract and the lower respiratory tract (LRT) [3].

*K. pneumoniae* pneumonia is a fatal disease with mortality rates of up to 22.7% [4], [5]. The incidence of *K. pneumoniae* pneumonia in the United States is more commonly associated with nosocomial acquisition of disease rather than environmental sources [6], [7], as *Klebsiella* is thought to contribute to only 1% of CAP in North America [8], [9], [10]. *K. pneumoniae* is the fifth most prevalent nosocomial bacterial pathogen in the United States for infections associated with UTIs, VAP and central line-associated bacteremia, and accounts for 6% of all nosocomial bacterial disease [11]. The emergence of *K. pneumoniae* as a nosocomial pathogen in the US and Europe may be due in part to the acquisition of antibiotic resistance markers providing a selective advantage in hospital settings, with particular concerns growing over an increasing prevalence of carbapenemase-expressing *K. pneumoniae* (KPC) strains [12]. Well characterized outbreaks of KPC dating back to 1988 have still not led to effective clinical diagnosis or control of these emerging pathogens in the US over 20 years later [13], [14]. Recently instated surveillance programs have begun to characterize the increasing threat of *K. pneumoniae* in the US health care system, where it is understood that the threat of *K. pneumoniae*, and in particular KPC, may be underestimated, particularly in long-term acute care facilities [15].

Several virulence determinants are important in mediating the virulence of *K. pneumoniae* in the lung, including capsular polysaccharide, lipopolysaccharide, enterobacterial common antigen, OmpA, OmpK36, the AcrAB efflux pump, the regulator RamA, the biofilm related factor YciI, and yersiniabactin [16], [17], [18], [19], [20], [21], [22], [23], however, additional undescribed systems may participate in *K. pneumoniae* virulence. To this end, numerous sequencing efforts have begun to characterize the genomes of *K. pneumoniae* strains including the first whole genome sequences of the clinical isolates MGH 78578 and NTUH-K2044 [24], and the more recent release of whole genome sequences of the strains HS11286 [25] and KCTC 2242 [26]. Comparative genetic analyses have successfully led to the identification and characterization of a novel allantoin metabolism locus required for GI tract disease which is present in the genome of NTUH-K2044, but absent from MGH 78578 [27].

The ATCC 43816 strain has been the focus of several studies characterizing the respiratory disease caused by *K. pneumoniae* [16], [18], [28], however this strain has not been sequenced, thus limiting investigations directed at identification of novel virulence determinants. To address this scientific gap, we have performed whole genome sequencing of ATCC 43816 to begin the characterization potential genomic differences relative to the sequenced *K. pneumoniae* strains.

We also decided to investigate host-pathogen interaction for the sequenced *K. pneumoniae* strains NTUH-K2044 and MGH 78578, for which little is known about their ability to cause respiratory disease or interact with professional phagocytes. We developed a novel pulmonary-specific delivery respiratory disease model in which to examine sequenced *K. pneumoniae* strains. Finally, we have characterized the ability of *K. pneumoniae* to modulate uptake and persist within multiple cultured murine macrophage cell lines.

## Materials and Methods

### Bacterial strains and media

*K. pneumoniae* strains were cultured routinely in Lennox Broth (LB) or LB agar plates at 37°C. *K. pneumoniae* was preconditioned for cell culture and animal studies by subculturing overnight broth cultures 1∶25 into TSBDC [29] for an additional 3 hr of growth at 37°C. Briefly, TSBDC is formulated as a concentrate of a 30 g/L trypticase soy broth mixed with 5 g/L of Chelex 100 in a 1/10^th^ volume, which is dialyzed from a 6–8 kDa dialysis tubing into a 1x volume of 1% glycerol, where the media consists of the small organic compounds which leave the dialysis tubing into the 1% glycerol solution. TSBDC is supplemented with 50 mM monosodium glutamate immediately prior to use. The bacterial cultures were washed into PBS and their concentration was estimated using OD_600_ measurements. The *K. pneumoniae* strains used in this study included ATCC 43816, MGH 78578 (kindly provided by Virginia Miller, UNC), NTUH-K2044 (kindly provided by Jin-Town Wang, NTUCM and Valley Stewart, UC Davis), and CIP 52.145 (Collection of Institut Pasteur). Where appropriate, antibiotics were used at the following concentrations unless otherwise stated: carbenicillin (100 µg/ml), kanamycin (25 µg/ml), zeocin (100 µg/ml), and gentamicin (20 µg/ml).

### Sequencing of *K. pneumoniae* ATCC 43816

ATCC 43816 genomic DNA was isolated from ∼5×10^9^ bacteria grown in LB broth overnight. The DNA was isolated in TE buffer with 0.5% SDS extraction in the presence of proteinase K and RNase, followed by phenol:chloroform:isoamyl alcohol (25∶24∶1 v/v) purification, and alcohol precipitation. A 1.5 µg aliquot of chromosomal DNA was processed for Illumina Next Generation Sequencing based on the manufacturer’s instructions. Briefly, fragmentase (NEB) was used to generate 100–300 bp DNA fragments which were end repaired, A-tailed, adaptor ligated, and PCR amplified using Phusion. Two lanes of 51 base reads were run to generate 22,422,915 reads of sequencing data, filtered to eliminate low quality reads, and assembled using Velvet [30]. We assembled 1763 contigs, of which 1550 contigs were >200 bp and were deposited at DDBJ/EMBL/GenBank under the accession APWN00000000. The version described in this paper is the first version, APWN01000000. The contigs were aligned against the non-redundant nucleotide database using BLASTN [31] and hits to the full genomes of the NTUH-K2044 and MGH 78578 strains were retained separately. Manual sorting was conducted to identify contigs common to or unique from the NTUH-K2044 and MGH 78578 genomes, and unique sequence was aligned by BLASTN to identify homology to other bacterial species.

The capsular polysaccharide genetic cluster was manually sequenced to close contig gaps between five contigs, as described elsewhere [32], and the complete sequence for the ATCC 43816 capsular polysaccharide locus have been deposited with DDBJ/EMBL/GenBank with the accession number KJ541664.

### Quantification of capsular polysaccharide production

Capsule production was quantified for *K. pneumoniae* from LB overnight cultures, as described elsewhere [33]. Briefly, PBS-washed bacteria were enumerated and subjected to hot phenol extraction before precipitating the chloroform-treated aqueous phase with 0.5 M sodium acetate and then 10 volumes of 95% ethanol. Polysaccharide was pelleted at 7200 g for 5 min after an overnight storage at −20°C. The pellet was resuspended in water, and uronic acid was measured from capsular polysaccharide preparations using a modified carbazole assay [34], with measurement calculated relative to a glucuronolactone standard.

### Generation of capsular polysaccharide mutants

Capsular polysaccharide mutants were generated for *K. pneumoniae* strains ATCC 43816 and NTUH-K2044 by allelic exchange mutagenesis by initially PCR-amplifying upstream (5′) and downstream (3′) 1 kb fragments from a gene targeted for knock out (Table 1). The 1 kb homologous fragments were assembled in pSK (Stratagene) using a HindIII restriction site common to both the upstream and downstream fragments. A HindIII floxed zeocin cassette was inserted between the upstream and downstream fragments before the assembled construct was moved into an allelic exchange vector, pJMW106, which is a Km^R^ variant of pCVD442 [35]. Thus, an XbaI-KpnI fragment containing an in-frame 89.3% coding region deletion of the NTUH-K2044 *wzc* gene was cloned into pJMW106, and electroporated into *E. coli* strain S17-1 [36] to yield strain S17-1/pJMW106-NTUH Δ*wzc*::flox-zeo. Similarly, a XhoI-SpeI fragment containing an in-frame 90.4% coding region deletion of the ATCC 43816 *manC* gene was used to generate the strain S17-1/pJMW106-ATCC Δ*manC*::flox-zeo.

**Table 1:** Primers used in this study.

**5′ ATCC ** ***manC*** ** XbaI(+)**	CTGCTCGAGATTACCAAAGATATCTTCACCAAGAAGGATGAAG
**5′ ATCC ** ***manC*** ** HindIII(−)**	GTAAAGCTTGCGAGACATCGGCCAGAGACGAC
**3′ ATCC ** ***manC*** ** HindIII(+)**	GAAAAGCTTGAGATCCAGTCGGGGTCGTACCTC
**3′ ATCC ** ***manC*** ** KpnI(−)**	GTGACTAGTTTTCGCTCCCGGCTGCTTCTGC
**ATCC ** ***manC*** ** mut(+)**	GTTATTCTACAATAAACTGACCAAGTCATCTTGTTTCCTCTCCTTCG
**ATCC ** ***manC*** ** mut(−)**	CTATCTTCCCGGGTTTCAGAAATTCGCCGTAGGC
**5′ NTUH ** ***wzc*** ** XbaI(+)**	CGTTCTAGAGCATAACGGTAAAGATACTAAGATCTCCTTATATGC
**5′ NTUH ** ***wzc*** ** HindIII(−)**	CAAAGCTTTATGATCAATAACTTCACCAATTAAACGACCTAGATCGATCC
**3′ NTUH ** ***wzc*** ** HindIII(+)**	CAAAGCTTTCGATGTTGCTAAAAATAGATTGGAACATAGCGGTGTTATAG
**3′ NTUH ** ***wzc*** ** KpnI(−)**	CTGGTACCTAATAATGAGGAGAACATTACCATAAAACGAGATGTATTTCG
**NTUH ** ***wzc*** ** mut(+)**	GGCAAAACTATGTTATTCGGACATTGGATAGGGCAACGAG
**NTUH ** ***wzc*** ** mut(−)**	CATTAATCGCAAGGCCAAATCCTTGTGATAATAGCATGCTTAGTATTC

Allelic exchange was conducted over two stages, first by bacterial conjugation of the allelic exchange vectors from S17-1 to *K. pneumoniae* and selection of Cb^R^Km^R^ merodiploid intermediates, and secondly by counter-selection of the suicide vector with 5% sucrose and zeocin. Confirmation of genome knock-out mutagenesis was confirmed on Km^S^ clones using PCR analysis with ‘mut’ primers (Table 1) which flank the deletion site. The resulting strains were named ATCC Δ*manC* and NTUH Δ*wzc*.

### Microscopic analysis of capsule mutants

Negative staining of *K. pneumoniae* capsule was conducted using nigrosin stain, as described elsewhere [37]. Briefly, LB overnight broth cultures of wild type and capsule mutant strains of ATCC 43816 and NTUH-K2044 were mixed 1∶1 with 10% nigrosin and smeared onto 18×18 mm coverslips. The smear was air dried before mounting onto a glass slide. Samples were visualized with a 63x objective on a Zeiss Axio microscope, and images were analyzed with Zeiss Axiovision Vs40x64 and Imaris x64 (Bitplane).

### Macrophage uptake assay

Both J774A.1 and RAW264.7 cell lines (ATCC) were cultured in DMEM (Invitrogen) supplemented with heat-inactivated 10% fetal bovine serum (FBS, HyClone) and seeded into 96 well microtiter plates at a density of 7.5×10^4^ or 2×10^5^ cells per well, respectively. Cells were challenged at an MOI of 10 with *K. pneumoniae* ATCC 43816, NTUH-K2044 or MGH 78578, or with capsule mutants ATCC Δ*manC* or NTUH Δ*wzc*. At one hour post infection, gentamicin was added to eliminate extracellular bacteria (20 µg/ml final, or 1000 µg/ml for the Gm^R^ MGH 78578 strain). Gentamicin concentrations were empirically determined to kill extracellular *K. pneumoniae* in DMEM/FBS at >99.99% efficiency within a 1 hr window. At three hours post infection, monolayers were washed with PBS, lysed with 0.1% Triton X-100/PBS for 5 min, and serially diluted for bacterial enumeration on LB plates. Statistical analysis of data sets was conducted by One-way ANOVA with Tukey post-test of log-transformed data.

### Macrophage survival assay

J774A.1 and RAW264.7 macrophages were cultured in 96 well microtiter plates as described above. Triplicate wells of macrophages were infected with *K. pneumoniae* in five replicate plates, and infections were conducted for 1 hr before the addition of gentamicin to kill extracellular bacteria, and antibiotic was maintained throughout the assay duration. At time points corresponding to 3, 4.5, 6, 9, and 12 hr post infection, a microtiter plate of samples was washed in PBS before releasing intracellular bacteria from macrophages using a 5 min treatment of 0.1% Triton X-100/PBS. Samples were serially diluted in PBS and plated onto LB plates to enumerate intracellular bacteria. Statistical analysis of growth between time point outgrowth was conducted by One-way ANOVA with Tukey post-test of log-transformed data.

### Intratracheal infection of mice

Murine infection studies were approved by the University of Louisville Institutional Animal Care and Use Committee in accordance with National Institutes of Health guidelines (Protocol #10069). Groups of five 8 wk old female BALB/c mice (Charles River) were challenged using a non-surgical intratracheal infection procedure was developed to minimize trauma during pathogen delivery. Intubation-mediated intratracheal (IMIT) inoculations were conducted as demonstrated in detail elsewhere [38]. Briefly, isoflurane-anesthetized animals received 10 µl of 2% lidocaine anesthetic to the rear of the throat and were supported supine on a tilting platform raised to a 45° angle. Using a fine tipped cotton applicator, the tongue was retracted while an otoscope fitted with a cut-away specula was inserted into the oral cavity to visualize the glottis. An 18 G catheter, cast with a silicone rubber sleeve (10 mm of catheter exposed) was used to intubate mice, using a 20 mil guide wire to assist catheter placement. A 20 G blunt needle was used to instill a 50 µl PBS bacterial suspension directly into the lung via the catheter, followed by a 150 µl volume of air to aid distribution of the inoculum. Infected animals were monitored twice daily for indications of moribund disease, at which point they were humanely euthanized by isoflurane. Studies were concluded at 14 days. Statistical analysis of survival data was conducted using Log-rank (Mantel-Cox) and Gehan-Breslow-Wilcoxon Tests (GraphPad Prism 5). Probit analysis (StatPlus 2009 Professional) was used to calculate the LD_50_ (Lethal Dose 50%) and both the upper (UCL) and lower (LCL) reliable interval values.

### Bacterial enumeration from key sites of infection

Groups of five BALB/c mice were infected using the IMIT model with 10^2.2^ CFU of either NTUH-K2044 or ATCC 43816. Moribund mice were euthanized at the presentation of lethargy, hunching, and labored breathing. Mice were euthanized by overdose of isoflurane, immediately followed by exsanguination by cardiac puncture with a 23 G needle, and the blood was collected to a Microtainer (K_2_EDTA, BD Biosciences). Lung, liver and spleen were each collected into a sterile Whirl-Pak bag (Nasco) and homogenized in 1 ml of sterile PBS, by rolling the tissue with a 25 ml serological pipette. Blood and tissue homogenate were subjected to detergent lysis with 1% Triton X-100 for 5 min and subsequently serially diluted in a 96 well plate. LB plates were spot-plated with 10 µl aliquots of diluted bacterial suspensions, grown for 8 hrs at 37°C, and bacterial burdens were calculated based on dilution factor, tissue weight, and estimated tissue density. Neutral buoyancy testing in glycerol solutions revealed that the estimated tissue densities for lung, liver, and spleen were 1.03, 1.08, and 1.06 g/ml, respectively.

## Results

### Whole genome sequencing of strain ATCC 43816

**Figure 1:**
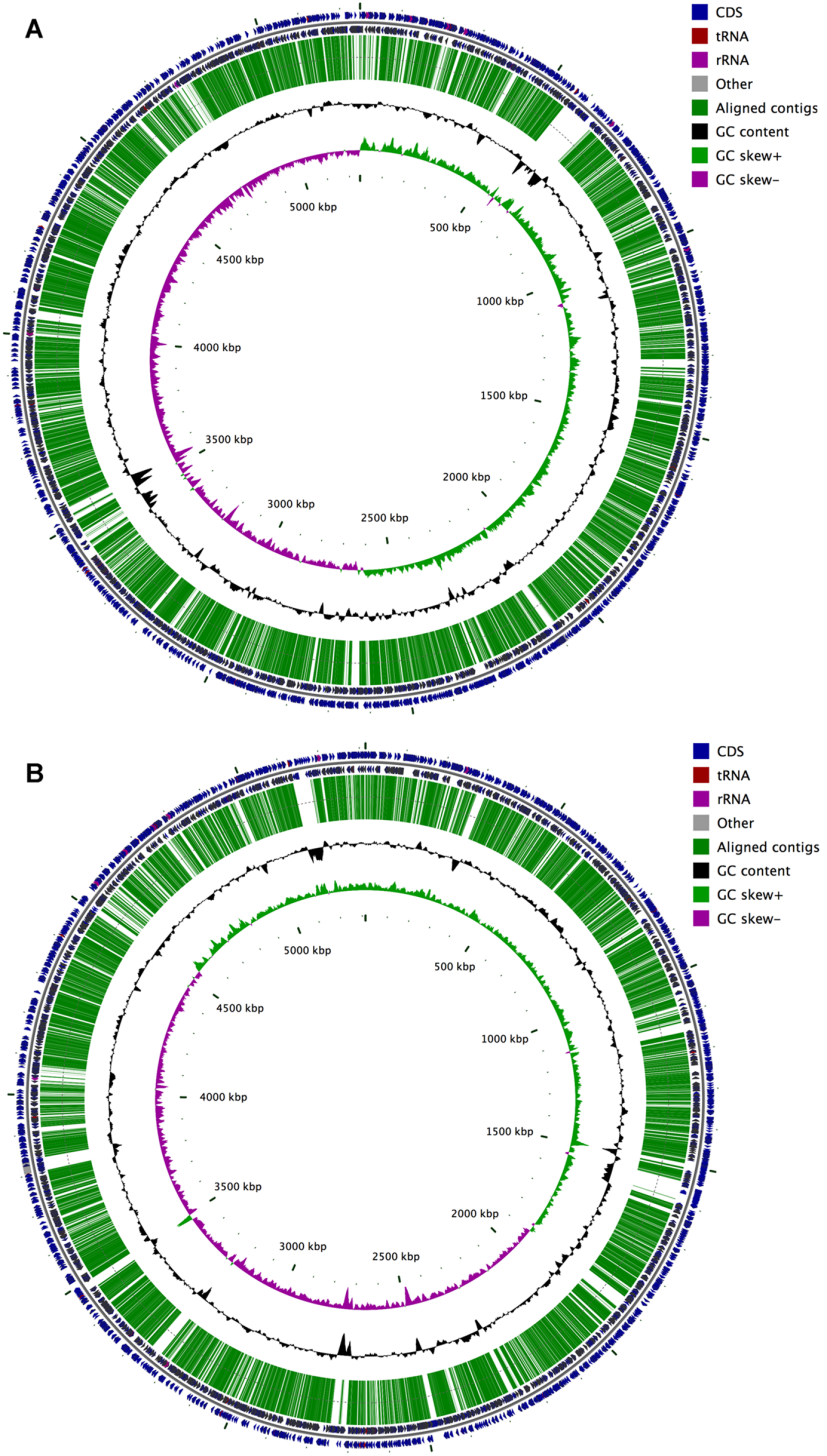
Alignment of the ATCC 43816 sequence to previously sequenced *K. pneumoniae* chromosomes. Next generation sequencing of ATCC 43816 produced 1763 contigs which were aligned to published NTUH-K2044 (Panel A) and MGH 78578 (Panel B) chromosomes. The locations of the contigs are shown as long green lines. The two outer tracks depict the annotated genes in the reference genomes (on both strands) while the two inner tracks show GC content and GC skew, respectively which strongly effects sequencing as can be seen from the coincidence of low GC content regions and gap in the contig alignments.

*K. pneumoniae* ATCC 43816 is a well-studied strain, capable of causing a moribund respiratory disease in mouse models. However, limited genomic data is available to support future investigations of *K. pneumoniae* pathogenesis, thus we decided to perform whole genome sequencing. Next Generation Sequencing was used to sequence the ATCC 43816 genome, which was subsequently assembled into 1763 contigs consisting of 4.207 MB of sequence data (accession number APWN00000000). To investigate the genetic relatedness of sequenced *K. pneumoniae* strains, the ATCC 43816 genome was aligned to the complete genome sequences of the NTUH-K2044 and MGH 78578 strains (Figure 1A and B, respectively). A total of 1676 contigs (4.128 MB) aligned with the NTUH-K2044 chromosome representing 78.7% genome coverage. Similarly, 1668 contigs (4.098 MB) mapped to 77.1% of the MGH 78578 genome, which includes two contigs mapping to the toxin-antitoxin system of the pKPN4 plasmid. We identified no additional sequence homology to *K. pneumoniae* plasmids, and the homologous toxin-antitoxin system is maintained on the chromosome for the NTUH-K2044 strain, suggesting that *K. pneumoniae* strain ATCC 43816 does not possess plasmids.

A high degree of genetic conservation was observed between the three *K. pneumoniae* strains with 96.3% of ATCC 43816 sequence mapping to both MGH 78578 and NTUH-K2044. However, 69.71 kb of ATCC 43816 contigs uniquely map with the NTUH-K2044 genome and 44.67 kb with the MGH 78578 genome (1.66% and 1.06% total sequence data, respectively). While MGH 78578 shares primarily metabolic and hypothetical proteins with ATCC 43816, NTUH-K2044 shares the virulence-associated yersiniabactin biosynthetic operon, a Type IV secretion system, an iron transport system, a CRISPR locus, and an acetonin catabolism locus (Table 2). Given the conservation of known virulence determinants between ATCC 43816 and NTUH-K2044, the data suggests that NTUH-K2044 may share the same virulence potential as ATCC 43816 in disease models. Conversely, MGH 78578 lacks several of the virulence determinants previously identified as critical to the disease potential of ATCC 43816 in murine respiratory disease models, suggesting that MGH 78578 may have a reduced virulence in these models, though MGH 78578 is notably a clinical lung isolate from a presentation of pneumonia.

**Table 2:** Genetic elements common/unique to sequenced strains.

Genetic loci common to NTUH-K2044	Genes mapped (KP1_)
Yersiniabactin biosynthesis	3583–3586, 3588–3593, 3605–3609, 3611–3613
Type IV Secretion	3634, 3638–3641, 3643
Iron transport	1980–1989
CRISPR locus	3164–3166, 3171
Acetonin catabolism	1112–1121
Conserved and hypothetical genes	2362–2364, 2378–2385, 3239–3240, 3773
**Genetic loci common to MGH 78578**	**Genes mapped (KPN_)**
Metabolic	00033–00034, 00594–00598, 03359–03370, 03372, 04612–04613
Conserved and hypothetical genes	01146–01148, 01151–01164, 01432–01433, 01316–01317, 04518
**ATCC 43816 genetic loci absent from NTUH-K2044 and MGH 78578**	**Genes**
*Escherichia coli* UMN026-like bacteriophage	ECUMN_0964–0965, 0975, 0977–0986, 0994–0996
*Klebsiella pneumoniae* subsp. *rhinoscleromatis* ATCC 13884-like bacteriophage	HMPREF0484_4775, 4777–4779, 1182
*Enterobacter radicincitans* DSM 16656 acriflavine resistance	Y71_5381–5385
*Klebsiella oxytoca* 10–5243 fimbrial biosynthesis	HMPREF9687_02419–02420
Hypothetical protein *Salmonella enterica* subsp. *enterica* serovar Saintpaul str. SARA29	SeSPB_A4698
Hypothetical protein *Enterobacter hormaechei* ATCC 49162	HMPREF9086_3347
Hypothetical protein *Klebsiella* sp. 4_1_44FAA	HMPREF1024_04074
Hypothetical protein *Escherichia hermannii* NBRC 105704	EH105704_01_06400

We also identified novel genetic sequences unique to ATCC 43816, and not present in either NTUH-K2044 or MGH 78578, notably including two bacteriophages, one of which is homologous to a bacteriophage found in the enteric *Escherichia coli* strain UMN026, and the other to the upper respiratory tract (URT) pathogen *Klebsiella rhinoscleromatis* strain ATCC 13884 (Table 2). Little has been reported regarding the clinical history of ATCC 43816, however the presence of both gastrointestinal (GI) and URT-related bacteriophages suggests that this *K. pneumoniae* strain may have previously been resident of both host niches.

**Figure 2:**
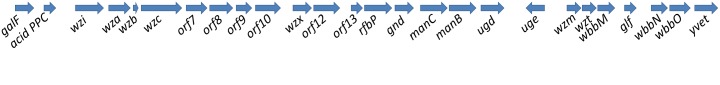
Genetic organization of the ATCC 43816 K2 capsule locus. Scale representation of the capsular polysaccharide biosynthetic locus (*galF-uge*) and LPS locus (*wzm-yvet*). The central domain of the capsule locus (*orf7-orf13*) represents the antigenic diversity region unique to K2 serotype capsules, while the remainder of the locus is well conserved with other *K. pneumoniae* capsule loci.

Next Generation Sequencing provided an estimated 78% coverage of the ATCC 43816 genome, however sequencing of the capsular polysaccharide biosynthetic locus was under-represented at 22% coverage (determined retrospectively). Given the importance of capsule as a virulence determinant, we completed sequencing of a 34.6 kb region which includes the capsule locus as well as an adjacent region (*wzm* to *wbbO*) reported to be required for LPS biosynthesis (Fig. 2) [39]. The K2 capsular polysaccharide locus shares broad homology to sequenced capsule loci from the *galF* to *wzc* and *rfbP* to *uge* genes, but shares specific homology over the central *orf7* to *orf13* genes to a subset of sequenced *K. pneumoniae* strains of K2 serotype. Homology over the entire capsule locus is therefore highest (99% identity) to that of the recently fully sequenced strains CG43 (Accession CP006648), KCTC 2242 (CP002910), and Kp52.145 (FO834906), and also to the partially sequenced capsule biosynthetic loci of VGH525 (Accession AB371296) and Chedid (D21242). The central region of the capsule loci encodes for the antigenic diversity of capsules, which has previously supported the use of PCR as a methodology to identify capsule serotype [40], [41].

### Characterization of capsule production

*K. pneumoniae* capsular serotypes influence the virulence potential of strains, as could the regulated production of capsule. Thus, we investigated whether differences exist in the amount of capsule produced by the three clinical strains examined in this study. *K. pneumoniae* were examined for their ability to produce capsule from LB overnight cultures. We found that ATCC 43816, NTUH-K2044 and MGH 78578 strains produced 9.7, 13.5, and 6.1 fg/CFU of capsule, respectively. As a control, we also measured capsule production from previously characterized strain 52.145 which produced 21.7 fg/CFU of capsule in our studies, consistent with 52.145 being a significant producer of capsule [33]. Our measurement of NTUH-K2044 capsule production is consistent with previously reported levels of 17.3 fg/CFU of capsule from overnight LB cultures [42]. Thus, of the sequenced strains studied in this work, NTUH-K2044 produced the greatest amount of capsule at levels 1.4 and 2.2 fold greater than ATCC 43816 and MGH 78578. Similar amounts of capsule were measured from strains grown to mid-exponential phase in TSBDC (data not shown), suggesting that media and growth phase do not significantly impact capsule production.

### Cell culture model

**Figure 3:**
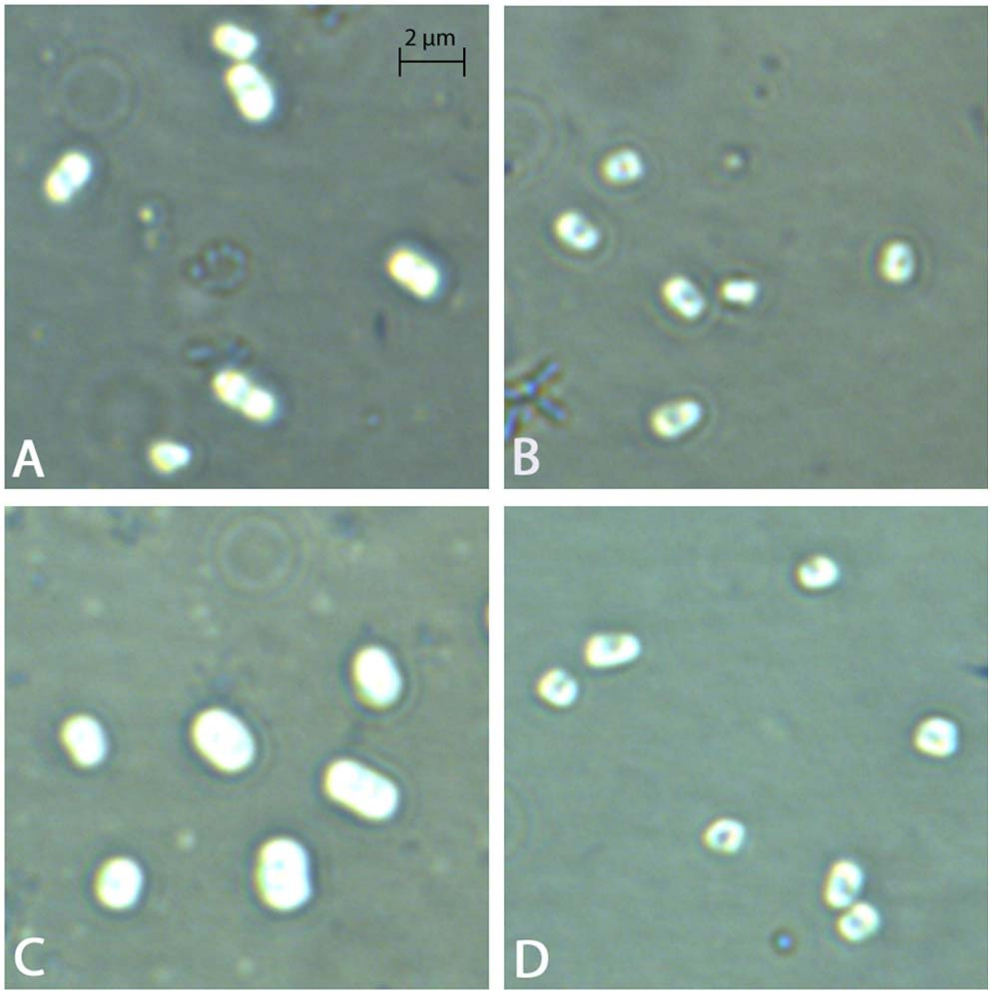
Negative staining of capsular polysaccharide from ATCC 43816 and NTUH-K2044 strains. Overnight bacterial suspensions of ATCC 43816 (A), ATCC Δ*manC* (B), NTUH-K2044 (C) and NTUH Δ*wzc* (D) were mixed in 1∶1 ratio with 10% nigrosin. The loss of the capsular polysaccharide from the mutant strains were illustrated by the decrease exclusion of the nigrosin dye which was visualized with Zeiss Axio microscope (63x magnification). Imaris analysis identified that the cross-sectional areas of ATCC Δ*manC,* and NTUH Δ*wzc* mutants were reduced by 36.21%, and 28.59%, relative to their isogenic parents.

**Figure 4:**
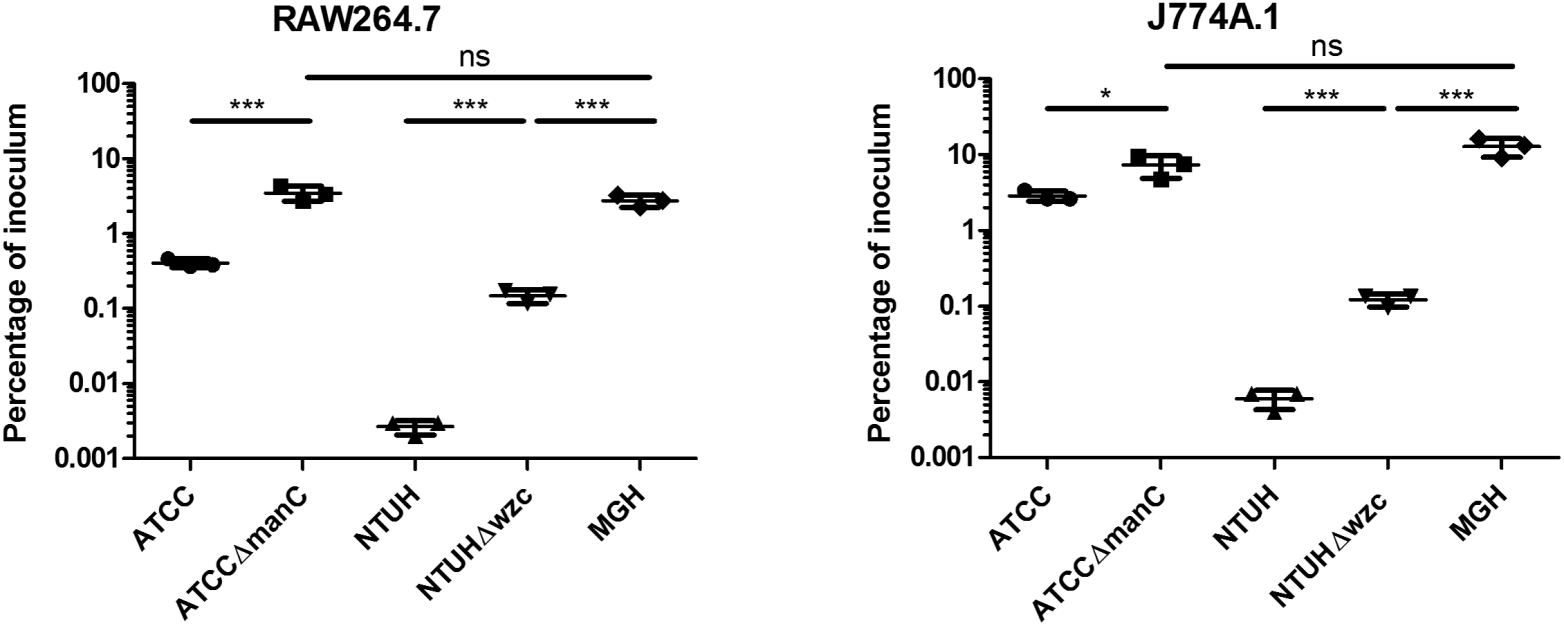
Uptake of *K. pneumoniae* wild type and capsular polysaccharide mutants strains into cultured murine macrophages. *K. pneumoniae* strains ATCC 43816 (K2), NTUH-K2044 (K1) and MGH 78578 (K52) or capsule mutants ATCC Δ*manC*, and NTUH Δ*wzc* were incubated in the presence of cultured murine macrophage cell lines J774A.1 or RAW264.7 at an MOI of 10 in 96 well plates. At one hour post-infection, gentamicin was introduced to eradicate extracellular bacteria, and uptake of *K. pneumoniae* strains into macrophages was assessed at 3 hr post-infection by plate counting. Triplicate samples were enumerated and data analyzed as a percentage of the inoculum, with the results representative of at least two independent trials.

*K. pneumoniae* is internalized by a variety of host cell types both *in* *vivo* and in cell culture models, thus we investigated whether the three study strains exhibit differences in uptake rates in cultured murine macrophages. Given that capsular polysaccharide has been reported to mediate an antiphagocytic phenotype, we also generated capsular polysaccharide mutants of ATCC 43816 (Δ*manC*) and NTUH-K2044 (Δ*wzc*), and confirmed that the capsule mutants exhibited reduced exclusion of nigrosin staining (Figure 3). Both J774A.1 and RAW264.7 monolayers were challenged with *K. pneumoniae* strains at an MOI of 10, and internalized bacteria were detected using a gentamicin protection assay. In both J774A.1 and RAW264.7 cells, the MGH 78578 strain was phagocytosed more efficiently than the ATCC 43816 and NTUH-K2044 strains (P<0.001) (Figure 4). This suggests that the K52 serotype capsule of the MGH 78578 strain does not resist uptake by murine macrophages to the same degree as representative K1 and K2 serotype strains. In addition, ATCC 43816 was phagocytosed more efficiently than NTUH-K2044 in both cell lines (P<0.001), suggesting that there may be differences in the antiphagocytic properties of K1 and K2 capsular polysaccharides or other surface exposed factors.

We therefore also investigated whether capsule alone mediates the antiphagocytic phenotype by comparing ATCC 43816 and NTUH-K2044 capsule mutants to relatively highly phagocytosed MGH 78578. Both ATCC Δ*manC* and NTUH Δ*wzc* capsule mutants were phagocytosed at significantly higher rates than their isogenic wild type parent strains in both cell lines (Figure 4), and furthermore, the NTUH Δ*wzc* was significantly less phagocytosed than the MGH 78578 strain. These data demonstrate that the NTUH Δ*wzc* capsule mutant retains antiphagocytic properties which are distinct from capsular polysaccharide, suggesting that additional factors additionally mediate the antiphagocytic phenotype of *K. pneumoniae*. The ATCC Δ*manC* was phagocytosed at levels similar to the MGH 78578 K52 strain, and it is therefore not possible to conclude whether ATCC 43816 possesses non-capsule antiphagocytic determinants using the K52 MGH 78578 strain which may itself be antiphagocytic. Due to the intrinsic antibiotic resistance of the MGH 78578 strain to common antibiotic markers used for molecular biology, we were unable to generate an acapsular MGH 78578 strain for these studies.

**Figure 5:**
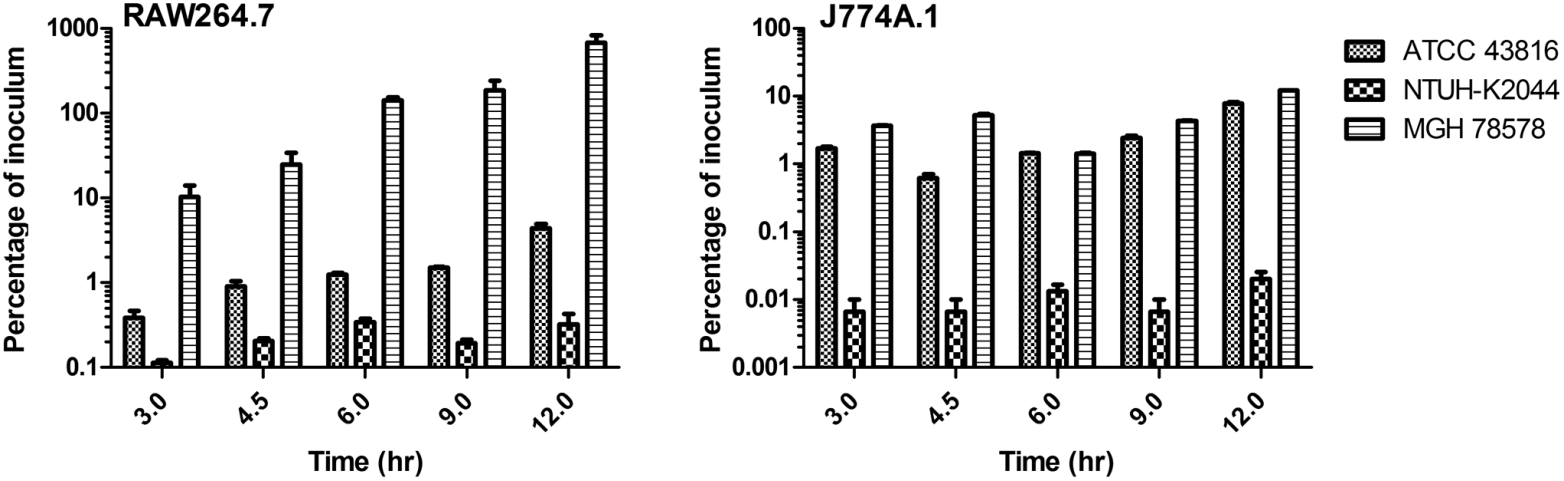
Growth potential of *K. pneumoniae* strains in cultured murine macrophages. *K. pneumoniae* strains ATCC 43816 (K2), NTUH-K2044 (K1) and MGH 78578 (K52) were incubated in the presence of cultured murine macrophage cell lines J774A.1 or RAW264.7 at an MOI of 10 in 96 well plates. At one hour post-infection, gentamicin was introduced to eradicate extracellular bacteria. At 3, 4.5, 6, 9, and 12 hr post-infection, a triplicate set of samples was processed for enumeration of intracellular bacteria. The data is representative of at least three independent trials.

We decided to investigate whether *K. pneumoniae* is replication-competent within cultured macrophages after internalization. Both RAW264.7 and J774A.1 murine macrophages were infected at an MOI of 10 with subsequent evaluation of bacterial colonization at time points 3, 4.5, 6, 9 and 12 hr post infection. As observed previously, MGH 78578 was internalized at the highest levels in both cell lines, while NTUH-K2044 had the lowest level of internalization at the 3 hr time point (Figure 5). Proliferation of *K. pneumoniae* was observed for all bacterial strains, however during 9 hr of observation between the 3 and 12 hr time points the average fold increase in bacterial number was just 3.0–4.6 fold in J774A.1 cells. Higher rates of proliferation were observed for ATCC 43816 and MGH 78578 in RAW264.7 cells, however NTUH-K2044 saw only a 2.9 fold increase in bacterial numbers from 3 hr to 12 hr. These data indicate that the K1 (NTUH-K2044) and K2 (ATCC 43816) strains of *K. pneumoniae* are internalized at relatively low rates but are replication competent in cultured macrophages. However, the K52 strain, MGH 78578, was phagocytosed at relatively high levels in both cell lines, and proliferated significantly in RAW264.7 cells. Importantly, intracellular ATCC 43816 and MGH 78578 exhibited significant outgrowth in cultured macrophages between the 3 and 12 hr time points (both J774A. 1 and RAW264.7, P<0.001), indicating that *K. pneumoniae* possesses replicative viability within macrophages, in spite of its classification as an extracellular pathogen. These data suggest that differences between *K. pneumoniae* serotype, and potentially other genetic determinants, could impact the preferred host niche during disease, including the propensity to persist within macrophages.

### Respiratory murine model of *K. pneumoniae* infection

**Figure 6:**
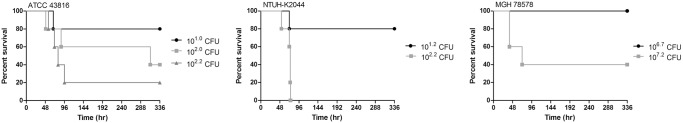
Survival analysis of *K. pneumoniae* respiratory challenge. Groups of five female BALB/c mice were challenged with *K. pneumoniae* strains ATCC 43816 (K2), NTUH-K2044 (K1) or MGH 78578 (K52) by IMIT respiratory infection. Mice were monitored for 14 days, and moribund mice were euthanized. The challenge dose for each survival curve group is indicated.

We decided to investigate the virulence potential of the three sequenced *K. pneumoniae* strains in a murine respiratory disease model. Because *K. pneumoniae* is known to colonize the upper respiratory tract (URT) in mammals, we developed a novel infection model to deliver *K. pneumoniae* non-surgically into the lung of mice using intubation-mediated intratracheal (IMIT) instillation, specifically modeling lower respiratory tract (LRT) disease. Female BALB/c mice were challenged with one of the three *K. pneumoniae* study strains using the IMIT infection method using multiple challenge doses to estimate the 50% lethal dose (LD_50_). Both ATCC 43816 and NTUH-K2044 were found to be highly virulent strains of *K. pneumoniae* (LD_50_<100) while the MGH 78578 strain is significantly less virulent, with an LD_50_>10^5.4^ fold higher than the virulent strains (Table 3). *K. pneumoniae* respiratory disease in the IMIT mouse model is associated with an acute course of disease with minimally lethal doses resulting in moribund disease within 3–4 days (Figure 6).

**Table 3:** Probit analysis of IMIT-infections of BALB/c mouse using *K. pneumoniae* strains.

Strain	LD_50_ (95% CI*_)_
ATCC 43816	4.71×10^1^ (1.36×10^1^−1.63×10^2^)
NTUH-K2044	2.33×10^1^ (5.4×10^0^−1.01×10^2^)
MGH 78578	1.37×10^7^ (9.50×10^6^−1.97×10^7^)

*95% confidence interval.

**Figure 7:**
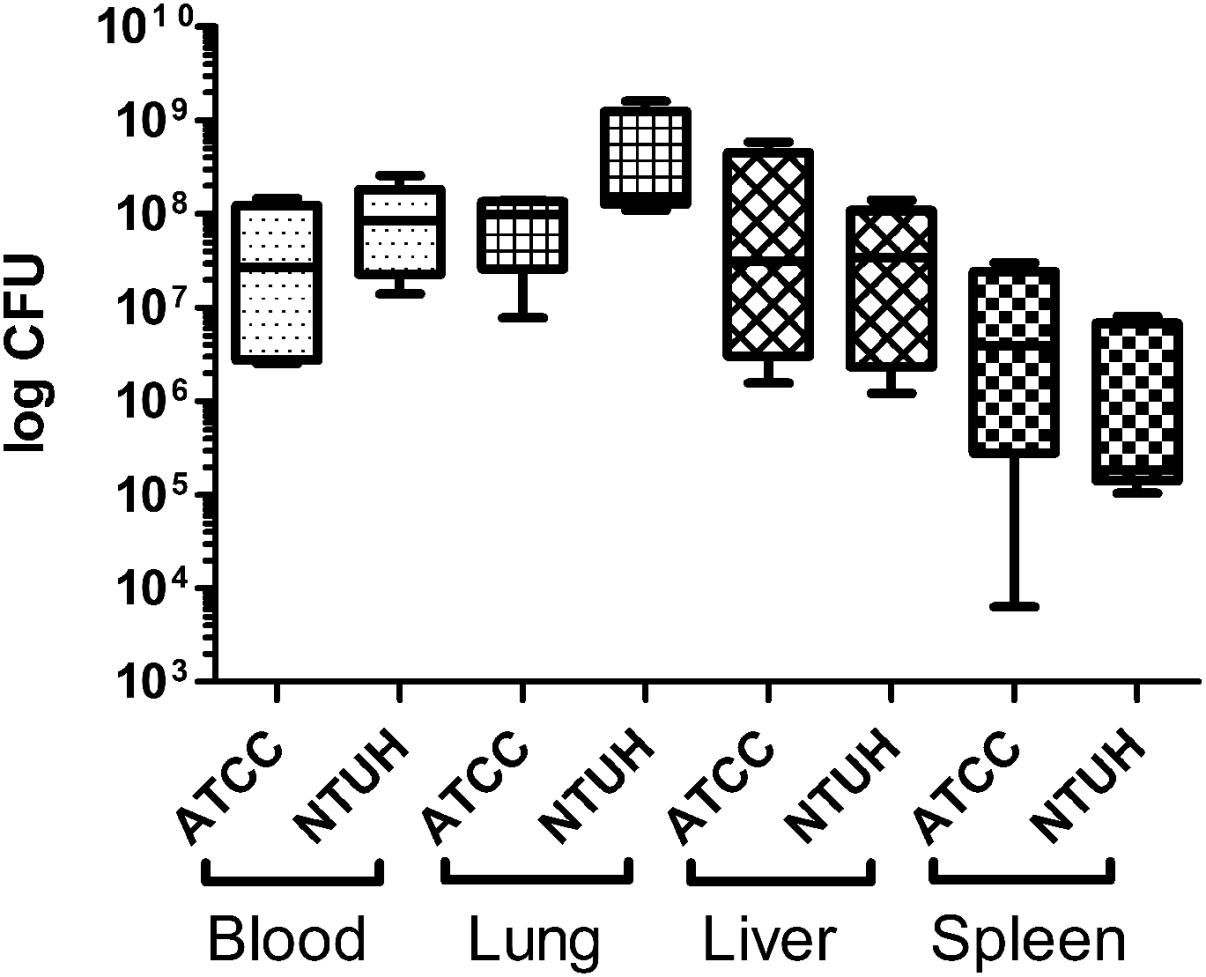
Bacterial burden of *K. pneumoniae*-infected mice. Groups of five female BALB/c mice were infected with *K. pneumoniae* strains ATCC 43816 or NTUH-K2044 and tissues were harvested at the onset of moribund disease, and homogenized in 1 ml of PBS Bacteria were enumerated from blood and from homogenates of lung, liver, and spleen. The results present a min/max box and whisker plot for each infected tissue (n = 5). Statistical analysis was carried out by one way ANOVA and Tukey post test.

Groups of mice infected with minimally lethal doses of ATCC 43816 and NTUH-K2044 (10^2.2^ CFU) were necropsied to investigate bacterial burdens in host tissues. Bacteria were enumerated by plate count from blood, and homogenates of lung, liver, and spleen. Moribund mice were found to have the highest levels of host colonization in both blood and lung, followed by liver and spleen (Figure 7). One-way ANOVA with Tukey Post Test revealed no significant difference between ATCC 43816 and NTUH-K2044 bacterial burdens in any of the host samples examined. These data reveal that both ATCC 43816 and NTUH-K2044 *K. pneumoniae* pneumonia is associated with development of a significant bacteremia and systemic spread to multiple organs.

## Discussion

*K. pneumoniae* respiratory infections may result from several mechanisms of pathogen introduction into susceptible hosts including inhalation of environmental sources of bacteria, as it relates to community acquired pneumonia (CAP), or nosocomial foreign body introduction of *K. pneumoniae* into the respiratory system, as in ventilator associated pneumonia (VAP). Thus, respiratory infections with *K. pneumoniae* are clinically important both to nosocomial pneumonia in hospital settings as well as CAP in developing areas of the world. The primary focus of this work was to gain insight into the molecular mechanisms which contribute to the respiratory disease caused by virulent *K. pneumoniae*. We therefore sequenced one of the commonly researched strains, capable of causing pneumonia in surrogate animal models. The ATCC 43816 strain was successfully sequenced by Next Generation approaches at approximately 80% coverage, based on estimated total sequence data relative to the NTUH-K2044 genome size. The majority of ATCC 43816 sequence (>96%) was well conserved to both the highly virulent NTUH-K2044 and the minimally virulent MGH78578 strains suggesting that *K. pneumoniae* strains may have large core genomes, and that key differences in virulence potential may be related to a small number of pathogenicity islands.

Based on the data from our murine respiratory disease model, we identified NTUH-K2044 and ATCC 43816 as highly virulent *K. pneumoniae* strains, and MGH 78578 as a low virulence strain. These findings are consistent with clinical evidence that K1 and K2 serotypes of *K. pneumoniae* are most commonly associated with severe disease presentations, including CAP, invasive presentations, as well as lethality in a mouse intravenous challenge model [7], [43]. To the best of our knowledge, this current study provides the first experimental evidence demonstrating that a representative K52 clinical isolate is relatively avirulent in a murine respiratory disease model. Thus, phenotypic evidence links the newly sequenced ATCC 43816 strain to the fully sequenced NTUH-K2044 rather than MGH 78578 strain. The genetic systems found to be shared between NTUH-K2044 and ATCC 43816, but absent from MGH 78578 included iron acquisition genes (yersiniabactin biosynthesis and iron transport), a Type 4 secretion system (T4SS), a CRISPR locus, and an acetonin catabolism locus. Yersiniabactin biosynthetic operon has been previously demonstrated to be important to the function of ATCC 43816 in a murine intranasal respiratory disease model [16], [17], where yersiniabactin is thought to contribute to evasion of the activity of lipocalin2 in the lung – a host factor which neutralizes enterobactin-based iron acquisition [44]. The T4SS present in the virulent ATCC 43816 and NTUH-K2044 strains has been proposed to potentially represent a DNA conjugation system, and is also present in the related *K. variicola* environmental isolate strain 342 [45], [46], thus future studies will be required to investigate the virulence potential of this secretion system in *K. pneumoniae*. This study supports previous findings that the capsular serotype and the presence of the yersiniabactin iron acquisition systems contribute significantly to the disease potential of highly virulent *K. pneumoniae* strains, with the additional possibility that T4SS or acetonin catabolism may be important for *K. pneumoniae* disease.

In this study, we investigated whether there were any significant differences in the ability of *K. pneumoniae* strains to persist within cultured macrophages. *K. pneumoniae* is considered to be primarily an extracellular pathogen, although there is evidence that this organism may be internalized in human epithelial cell lines [47], [48]. Furthermore, *K. pneumoniae* can be taken up into cultured murine peritoneal macrophages, and are also internalized *in* *vivo* in alveolar macrophages [49]. However, capsular polysaccharide may mediate blocking the initial attachment of bacteria to cells, reducing internalization [50]. Given the importance of capsule in mediating the uptake of *K. pneumoniae* into phagocytic cells, we anticipated that the representative K2 strain in our study, ATCC 43816, may resist uptake into cultured macrophages given the previous discovery that the K2 serotype is associated with an absence of mannose residues in the capsular polysaccharide [51]. Similarly, the *gmd* and *wcaG* genes, present in K1 serotype capsular loci, are required for the modification of mannose to fucose, and a corresponding low level of mannose/high level fucose in *K. pneumoniae* isolates which possess these genes [52]. We had observed that the K1 strain NTUH-K2044 exhibited particularly low uptake into cultured murine macrophages in our studies, and that the K2 strain ATCC 43816 also had a lower level of uptake than the K52 strain MGH78578. Similarly, capsule has been demonstrated to mediate anti-phagocytosis in both amoeba and alveolar macrophages, suggesting that this role for capsule is ubiquitously important across a range of host-pathogen interactions with professional phagocytes [21], [53]. Given the potential role of the mannose receptor in facilitating phagocytosis, and the reduced level of mannose residues in K1 and K2 serotype capsules, it is possible that modulation of the polysaccharide surface of *K. pneumoniae* may represent an important strategy for evading host defense.

Consistent with prior studies, we found that acapsular *K. pneumoniae* mutants also exhibited increased uptake into macrophages, however, we also observed that the increase in uptake of the NTUH-K2044 capsule mutant did not achieve the level of uptake of the representative K52 serotype strain. This finding suggests that the NTUH-K2044 strain possesses additional non-capsule antiphagocytic factors, or that the MGH78578 strain actively promotes its uptake into macrophages. Given that *K. pneumoniae* capsule does possess antiphagocytic properties as a strategy to act primarily as an extracellular pathogen, we hypothesize that the K52 MGH 78578 strain does not promote its own uptake, and instead, we hypothesize that the function of K52 capsules would be as an antiphagocytic determinant consistent with other serotype capsules. Thus, we interpret that the NTUH-K2044 strain possesses multiple mechanisms to resist phagocytosis, which is consistent with previous findings that capsule, LPS, carnitine metabolism, and the ClpX protease are all required to resist entry into amoeba and human neutrophils [53]. We conclude that *K. pneumoniae* antiphagocytosis is mediated by a multifactorial process and that capsule alone is insufficient to account for this phenotype. This conclusion is consistent with prior published findings that additional genetic loci participate in mediating *K. pneumoniae* antiphagocytosis, including genes for LPS, the ClpX protease, and carnitine metabolism [53].

In our respiratory challenge studies, the K1 and K2 serotype strains possessed a significantly higher virulence in mice than the K52 serotype strain, supporting the possibility that evasion of phagocytosis is a strategy employed by *K. pneumoniae* to enhance virulence in mammalian hosts. Thus, these finding support the characterization of *K. pneumoniae* as an extracellular pathogen, whereby the most virulent strains are also the most antiphagocytic. These results are consistent with previous reports which have demonstrated that antiphagocytic properties of *K. pneumoniae* capsule are associated with highly virulent K1 and K2 serotypes in panels of Asian strain isolates [54], [55]. It is however noteworthy that several additional virulence-associated genetic determinants are common to the K1 and K2 representative strains investigated in this study, including mechanisms of iron acquisition, highlighting that *K. pneumoniae* serotype is not the sole distinguishing factor for predicting disease potential in mammalian hosts.

Our data supports a link between evasion of phagocytosis and virulence potential as an interpretation that *K. pneumoniae* is primarily an extracellular pathogen. However, we interestingly demonstrate that *K. pneumoniae* strains are fit to replicate within macrophages once internalized. This is an intriguing finding that may suggest that a subpopulation of *K. pneumoniae* may use an intracellular lifestyle as part of the disease process. Future studies will be required to characterize to what extent *K. pneumoniae* has adapted to intracellular host-pathogen interaction, and what role the intracellular lifestyle might play *in* *vivo*.

We developed a novel respiratory disease model to study *K. pneumoniae* pneumonia which facilitated an investigation of the lower respiratory tract (LRT) colonization in the absence of the involvement of other primary sites of infection. Our motivation to develop this model system was in part shaped by our observations that bacterial pathogens may opportunistically infect the upper respiratory tract (URT) in a process which is unique from that observed during human disease [56]. Direct non-surgical instillation directly into the LRT also directly mimics a normal route of nosocomial acquisition of *K. pneumoniae* associated with VAP. Our novel intubation-mediated intratracheal (IMIT) delivery of bacteria facilitates: i) the use of a flow meter to validate the placement of a catheter into the trachea, ii) the ability to instill bacteria directly into the lung via a blunt needle inserted through the catheter lumen, iii) delivery of bacteria into the lower lobes of the lung under positive pressure, and iv) minimal trauma to the host owing to the non-surgical nature of the procedure.

We found that our novel IMIT infection model lowered previously reported LD_50_ values for both i.n. and surgical i.t. infection models, suggesting that targeted delivery of a reduced number of bacteria to the lungs enhances the disease potential of *K. pneumoniae*. The LD_50_ for the ATCC 43816 strain in the IMIT model is 10^1.8^–10^2.8^ fold less than previously published LD_50_ values using intranasal models [16], [57], [58], suggesting that the IMIT model provides a lower LD_50_ due to the ability of the intubation tubing to briefly occlude the air space, allowing for positive pressure delivery of organisms deep into the lung, rather than passive delivery into the lung in shallowly breathing anesthetized animals. Thus, our IMIT model improves the disease potential of *K. pneumoniae* in lung-specific disease, and may be an excellent instillation method for therapeutic and diagnostic reagents in studies requiring LRT delivery.

In summary, our study has provided a first draft sequence of the ATCC 43816 genome which will support future investigations of *K. pneumoniae* function. We also have provided the first description of the growth potential of *K. pneumoniae* in cultured murine macrophages, supporting a growing body of evidence that *K. pneumoniae* may not be exclusively an extracellular pathogen. Finally, we have developed and employed a novel non-surgical, lung-specific infection model which allows for targeted low dose inoculation of *K. pneumoniae* giving rise to a lethal pneumonia in mice, and revealing a significant difference in the disease potential of clinical isolates.
